# Report on the seventh Japanese meeting on biological function and evolution through interactions between hosts and transposable elements

**DOI:** 10.1186/s13100-025-00392-7

**Published:** 2026-01-30

**Authors:** Kenji Ichiyanagi, Yoko Ikeda, Kuniaki Saito

**Affiliations:** 1 Laboratory of Genome and Epigenome Dynamics, Department of Animal Sciences, Graduate School of Bioagricultural Sciences, Nagoya University, Furo-cho, Chikusa-ku, Nagoya, 464-8601 Japan; 2 Institute of Plant Science and Resources, Okayama University, Chuo 2-20-1, Kurashiki, 710-0046 Japan; 3 Invertebrate Genetics Laboratory, National Institute of Genetics, Yata 1111, Mishima, Shizuoka, 411-8540 Japan

**Keywords:** Transposon, Retrotransposon, Epigenetics, Transposition, Functionalization

## Abstract

The seventh Japanese meeting on host–transposon interactions, titled “Biological Function and Evolution through Interactions between Hosts and Transposable Elements,” was held on September 1st and 2nd, 2025, at the National Institute of Genetics, as well as online. This meeting was supported by the National Institute of Genetics and aimed to bring together researchers studying the diverse roles of transposable elements (TEs) in genome function and evolution, as well as host defense systems against TE mobility, TE bursts during evolution, and intron mobility in mammals, insects, land plants, fungi, and protozoa. Here, we present the highlights of these discussions.

## Introduction

Evidence suggests that transposable elements (TEs) play key roles in biological processes. The interactions between TEs and their hosts were discussed in a series of meetings titled “Biological Function and Evolution through Interactions between Hosts and Transposable Elements” [1–4]. This event was held at the National Institute of Genetics (NIG) in Mishima, Japan, where biologists studying TEs in different species gathered and exchanged ideas. At the seventh meeting, held on September 1st and 2nd, 2025, we invited 17 speakers to present their recent work in seven sessions chaired by Kei Fukuda (Yamanashi University, Japan), Jun-ichi Nakayama (National Institute for Basic Biology, Japan), Yasuo Ariumi (Nagasaki University, Japan), Eriko Sasaki (Kyushu University, Japan), Atsuko Shirai (RIKEN, Japan), Mikiko Siomi (University of Tokyo, Japan), and Yuka Iwasaki (RIKEN, Japan). The sessions included approximately 50 onsite attendees and 25 online attendees.

### Highlights of the talks

#### Special lecture

At lunch time on the second day, Tetsuji Kakutani (National Institute of Genetics, Japan) presented a special lecture on the epigenetic silencing of TEs in the model plant *Arabidopsis thaliana* [5, 6] and on anti-silencing systems mediated by proteins named VANCs [7–9]. He presented unpublished results on the structure and interaction of VANCs and their target DNA, which suggest coevolution of the protein structure and its target DNA sequence. He also discussed the role of tandem repeats and TEs in the rapid evolution of sequence-specific anti-silencing systems as well as centromeres [10–12].

#### TE diversity

 Kenji K. Kojima (Genetic Information Research Institute, USA) introduced the diversity and current TE classification scheme within the framework of the eukaryotic repeat database RepBase. The rapid accumulation of genome sequence data has expanded our knowledge of TE diversity, but simultaneously obscures the boundaries between TEs and other selfish genetic entities, such as viruses and homing endonuclease genes. *Polinton*-like viruses, for instance, challenge our understanding of TEs because they behave like TEs and also like viruses. Beyond the Tree of Life, some eukaryotic DNA transposon superfamilies share certain characteristics with prokaryotic insertion sequences (ISs). *IS481EU* is a new addition to such eukaryotic superfamilies, showing similarities to the prokaryotic IS*481* family [13]. Retroelements, which encode a reverse transcriptase, also include a new group, *Troyka*, as an independent lineage of long terminal repeat (LTR) retrotransposons [14]. Newly discovered composite non-autonomous LTR retrotransposons *Helenus* and *Ajax* use small RNA-derived sequences for transcription, analogous to short interspersed elements (SINEs) [15]. Horizontal transfer and modularity appear to be the key to understand TE diversity.

Toshiharu Ishikawa (Nagoya University, Japan), a graduate student in the Ichiyanagi Laboratory, presented recent findings on the genome of an insect of Pseudococcidae family (mealybug). Interestingly, its sex is determined by paternal genome silencing, in which the paternal chromosomes are heterochromatinized during early embryogenesis to become males, whereas females keep all chromosomes in an active state [16]. He and his colleagues constructed its genomic sequence at the chromosomal level, which revealed that 43% of the genome is comprised of repetitive sequences, with DNA transposons being particularly abundant (28.7%). Sequence divergence of these DNA transposons suggests three major bursts of amplification, with the latest burst apparently still ongoing, implying that TE activity is currently generating diversity in the population.

#### Mechanisms and regulation of transposition

It is important, not only from the aspect of basic biology, but also from biotechnology aspects, to understand molecular mechanisms of transposition reactions. Tomoichiro Miyoshi (RIKEN, Japan) reported recent findings on how LINE and SINE retrotransposition is regulated by host factors. Human LINE-1 encodes two proteins, ORF1p and ORF2p, both of which are required for LINE-1 retrotransposition. He previously reported that LINE-1 exploits host DNA repair factors to facilitate its mobilization [17]. However, host cells have developed anti-retrotransposon systems to maintain genome integrity [18, 19]. Despite extensive studies on LINE-1 regulators, the host factors that control Alu, a human SINE family, remain poorly understood, largely due to the lack of suitable tools to monitor Alu mobilization. To expand the repertoire of Alu retrotransposition reporters and explore its regulation, he and his colleagues developed an EGFP-based fluorescent reporter for Alu retrotransposition. EGFP-positive cells obtained with this new reporter exhibited Alu insertions displaying the hallmarks of canonical retrotransposition, including target-site duplication and the LINE-1 endonuclease cleavage site. Furthermore, they demonstrated that this reporter can be adapted for other SINE families, such as mouse B2 elements. This system enables the detection of Alu activity using flow cytometry and live-cell imaging without antibiotic selection.

Sayuri Tsukahara (The University of Tokyo, Japan) presented her research on the integration mechanism of the centrophilic retrotransposon *Tal1*, an LTR retrotransposon in *Arabidopsis lyrata*. She and her colleagues previously showed that the introduction of *Tal1* into the *A. thaliana* genome resulted in *de novo* integration into centromeric satellite repeat regions [20]. Their recent study described that *Tal1* integrates into regions occupied by CENH3 (centromere-specific histone H3), and that ectopic expansion of the CENH3-occupied region results in the spread of *Tal1* integration regions, suggesting that *Tal1* targets CENH3-containing chromatin [12]. They also demonstrated that the C-terminal region of *Tal1* integrase was responsible for targeting centromeres, as a single amino acid substitution within this region disrupted centromere-specific integration of *Tal1*. She also presented unpublished results regarding additional structural variations controlling the targeting properties of *Tal1*.

 Hiroshi Nishimasu (The University of Tokyo, Japan), a structural biologist working on CRISPR-Cas systems and nucleases [21–24], presented his recent studies on the transposase of bacterial IS621, a DNA transposon [21]. IS621 is intriguing in that, when circulized, it generates a non-coding RNA called a bridge RNA, which directs transposase activity. The bridge RNA consists of a target-binding loop and a donor-binding loop, and binds to the transposase. The cryo-EM structure revealed that the transposase forms a dimer of dimers, with one dimer binding to the target-binding loop and the other to the donor-binding loop. Active sites are formed between the two dimers and positioned at the recombination site of the donor and target DNA. The top strands of the target and donor DNA are cleaved at these active sites, after which the cleaved DNA strands are exchanged and re-ligated, resulting in a Holliday junction intermediate. This intermediate is then resolved by cleavage of the bottom strands. Overall, the IS621 complex structure reveals how the bridge RNA confers target/donor specificity to the transposase. Since the guide segments in the bridge RNA can be altered, the IS621 system enables programmable recombination between various DNA molecules for genome engineering. Therefore, the IS621 structure provides a framework for the engineering of the bridge system with enhanced activity and specificity.

#### Epigenetic regulation of TEs

Epigenetic mechanisms, including DNA methylation, histone modification, and PIWI-interacting RNAs (piRNAs), play important roles in regulating TE activity at both transcriptional and post-transcriptional levels. It is widely accepted that heterochromatin is a critical component of chromosomes marked by repressive epigenetic marks. While the formation of locus-specific heterochromatin in mammals is being increasingly understood, the mechanism guiding it in plants remains obscure, as the sequence-driven DNA methylation mechanism in plants functions mainly within euchromatic regions. Taiko Kim To (Institute of Science, Tokyo, Japan) and her colleagues previously identified a novel mechanism for heterochromatin formation that is independent of the well-known *de novo* DNA methylation mechanism, RdDM [25–27]. However, it remains unclear how this mechanism targets the regions that should undergo heterochromatin silencing. At the meeting, she presented their recent data on extensive epigenomic analyses, emphasizing the importance of histone H2A variants, H2A.Z and H2A.W, in *de novo* heterochromatin formation [28].

Takuji Tsukiyama (Kindai University, Japan) studied *Miniature Ping* (*mPing*), which is the first active miniature inverted-repeat TE (MITE) identified in the rice genome and an excellent model for studying how MITEs proliferate to tens of thousands in the host genome [29]. The DNA transposon *Ping* provides two proteins (Myb-like protein and transposase) for *mPing* transposition. It has been believed that *Ping* successfully escaped host-mediated transcriptional silencing because the expression of *Ping* was detected in both *mPing*-active and -inactive rice strains [30]. He presented unpublished results indicating that *Ping* is a target for host-mediated transcriptional silencing in several rice cultivars. Intriguingly, this silencing does not appear to involve DNA methylation. How *Ping* is silenced remains to be elucidated.

Soichiro Yamanaka (Hamamatsu University School of Medicine, Japan) introduced his study on the epigenetic regulation of TEs in mouse germ cells. He and his colleagues discovered that chromatin at TE loci becomes open and transcriptionally active in male fetal germ cells. They proposed that this transient chromatin relaxation facilitates genome-wide *de novo* DNA methylation during the developmental window [31]. However, persistent activation can trigger germ cell apoptosis and infertility. Of note, they found that transiently activated TEs are subsequently repressed through MORC1-dependent H3K9me3 deposition [32]. In contrast, only a subset of transposons is activated in female fetal germ cells. Therefore, transient TE activation occurs in both male and female germ cells, but the modes of their activation are sexually dimorphic, highlighting germ cells as critical arenas for host–transposon interactions over evolutionary timescales.

To advance our understanding of piRNA-directed transposon silencing, Taichiro Iki (Osaka University, Japan) and his colleagues adapted TurboID, an in vivo proximity biotin-labeling technique, to the insect model *Drosophila melanogaster*. They successfully employed this method in testicular germ cells to catalogue the client proteins of the testis-specific molecular chaperone Cyclophilin 40 (Cyp40/PPID) [33, 34], and to characterize the cofactors of the sex chromosome regulator Ctr9, enriched in the testis (Ctr9t) [35]. TurboID was then employed in female germ cells to define the proximity proteome of PIWI-clade Argonaute family proteins (Piwi, Aubergine, and Ago3), which are the essential partners of piRNAs and central effectors of transposon silencing. Germline PIWI-TurboID analysis first recovered a set of known piRNA pathway components, demonstrating that the proteome reflects functional proximity. Moreover, RNAi-based knockdown screening identified several novel candidates involved in the piRNA pathway and transposon silencing. By integrating transcriptome analysis, piRNA sequencing, and Cut&Tag-based chromatin profiling, the researchers are now elucidating the roles of Piwi proximity factors in controlling transposons that contribute to telomere formation.

Rippei Hayashi (Australian National University, Australia) presented recent findings on the co-evolution of endogenous retroviruses (ERVs) and host defense mechanisms. It has been previously postulated that *Ty3/gypsy* LTR retrotransposons acquired an *envelope* gene during the recent evolutionary history of *Drosophila*. These *envelope*-carrying *gypsy* elements are active in the somatic cells of the ovaries and can infect female germline cells. *Drosophila* species have developed defense mechanisms based on the Piwi-interacting RNA (piRNA) pathway to counter their activities. Contrary to this established model, a recent study showed that *envelope*-carrying *gypsy* elements are widespread in metazoan species, including ancient animals such as cnidarians, ctenophores, and tunicates, suggesting that their origin dates back to before the split of bilaterian and non-bilaterian animals in the Pre-Cambrian era [36]. Furthermore, they found that the piRNA pathway that targets the *envelope*-carrying *gypsy* in the native niche of ovarian somatic cells is widely conserved across insects, indicating a long-term evolutionary arms race.

Hitoshi Nakayashiki (Kobe University, Japan) has studied the relationships among TE amplification, DNA methylation, and DNA double-strand breaks in the fungus *Pyricularia oryzae* [37]. He introduced a study showing that the copy number and DNA methylation levels of the MAGGY retrotransposon increased upon introduction of MAGGY into a MAGGY-less strain. Rad51, Rad55, and Rad57 mediate either the overall level or the copy number-dependence of de novo DNA methylation, likely in conjunction with DNA repair. Interestingly, mutations in DCL1 and AGO2, which are involved in the RNAi pathway, impair copy number-dependence of MAGGY methylation. Co-immunoprecipitation experiments suggested a physical interaction between RNAi and homologous recombination to inhibit the uncontroled amplification of this retrotransposon.

Kensuke Kataoka (National Institute for Basic Biology, Japan) presented his recent findings on the heterochromatin-mediated process of TE silencing in the ciliate *Tetrahymena.* While heterochromatin plays a central role in TE silencing in eukaryotes, the unicellular eukaryote *Tetrahymena* goes one step further—eliminating heterochromatinized TEs from its active genome. Similar to other ciliates, *Tetrahymena* possesses two types of nuclei within a single cell: the transcriptionally inactive germline micronucleus (MIC) and the transcriptionally active somatic macronucleus (MAC). During sexual reproduction, the MIC undergoes meiosis and generates a new MAC for the next generation. During MAC differentiation, tens of thousands of transposon loci in the genome are marked by histone methylation (H3K9me3 and H3K27me3) via a small RNA-mediated pathway and are ultimately eliminated from the MAC genome [38]. Kataoka showed that members of the Heterochromatin Protein 1 (HP1)-like proteins, which recognize H3K9me3 and H3K27me3 [39, 40], interact with one another to cluster TE loci into a limited number of nuclear condensates. The researchers further characterized the roles of each HP1-like protein in condensate formation and found that one of the HP1-like proteins played a central role in condensate formation through phase separation, whereas the others fine-tuned the dynamics of the resulting condensate. This cooperative role of HP1-like proteins likely confines the potentially deleterious DNA elimination process to clustered heterochromatin, thereby ensuring the faithful and robust removal of TEs.

#### Interactions between host function and TEs

Accumulating evidence suggests that TEs are activated in the human brain and play a role in the pathogenesis of neurological disorders. Therefore, it is important to understand the epigenetic features of TEs and inter-individual genetic diversity caused by TE transposition in the human brain. Risa Watanabe (Icahn School of Medicine at Mount Sinai, USA) shared their findings on the genetic and epigenetic heterogeneity of TEs in the postmortem human brain. She and colleagues utilized Fiber-seq, a single-molecule long-read sequencing method that maps the primary architecture of multikilobase chromatin fibers at nucleotide resolution​ [41]. Across 61 samples, approximately 2,000 and 1,000 TE-related insertions (INS) and deletions (DEL), respectively, were detected. Half of the TE-INS were intragenic and enriched in neural genes. Interestingly, Alu and LINE-1 elements exhibited nucleosome phasing in their adjacent sequences. They also revealed a discrepancy between Fiber-seq and ATAC-seq in LINE-1 promoter accessibility profile in the brain, suggesting the importance of utilizing long-read methods over conventional short-read methods for studying TE accessibility.

Human genes harbor many TEs in their introns; however, the roles of intronic TEs remain largely unknown. Yasunori Aizawa (Institute of Science Tokyo, Japan) revealed how intronic Alu elements suppress gene expression by using their genome engineering method called UKiS (universal knock-in system) [42]. They precisely removed all Alu copies from the first intron of the *TP53* gene locus, resulting in a sixfold increase in mRNA and a threefold increase at the protein level. Interestingly, removal of intronic Alu clusters from two other human genes did not affect the transcriptional activity of their respective parental genes. These results imply the presence of an unknown mechanism where Alu retrotransposition events into *TP53* intron modulated its gene expression during primate evolution.

Environmental stress often activates TEs, and this transcriptional activation may play a role in the stress response. One such example is *ONSEN*, a heat-activated LTR retrotransposon found in plants [43]. Hidetaka Ito (Hokkaido University, Japan) described stress-induced TEs, including *ONSEN*, which is transcriptionally activated by heat shock factors (HSFs), the pivotal transcription factors during heat stress. Environmental temperature affects the activity of *ONSEN*, as revealed by the analysis of various ecotypes of *Arabidopsis thaliana*, which showed that the copy number of *ONSEN* is higher in ecotypes living in regions with greater temperature amplitude. Other types of stress, including biotic interactions, can also influence TE activity. Under these conditions, plants activate stress-related transcription factors, leading to the transcriptional induction of several TEs, including *ONSEN*. These findings underscore the close relationship between environmental stress and TE dynamics [44]. *ONSEN* has been shown to preferentially retrotranspose into genic regions, suggesting that stress-induced activation of this TE may contribute to phenotypic diversity and environmental adaptation.

Takahito Takei (National Institute of Advanced Industrial Science and Technology, Japan) shared his recent findings on how the RNA-directed DNA methylation (RdDM) pathway contributes to gene expression control by regulating TEs. He focused on PDS5C and PDS5E, non-canonical components associated with the RdDM pathway, and reported that loss-of-function mutants of these factors exhibit pronounced phenotypic changes accompanied by de-repression of TEs located near protein-coding genes [45]. Using epidermal development as a model, he showed that PDS5C/E-mediated TE regulation plays a critical role in controlling the expression of nearby genes. This work suggests an unconventional mechanism of gene regulation distinct from the canonical transcriptional repression mediated by RdDM. Future studies are expected to identify all the genes subject to regulatory mechanism and clarify their conservation across plant species.

## Data Availability

No datasets were generated or analysed during the current study.

## Abbreviations

TEs: Transposable elements
NIG: National Institute of Genetics
ISs: Insertion sequences
LTR: Long terminal repeat
SINEs: Short interspersed elements
piRNAs: PIWI-interacting RNAs
MITE: Miniature inverted-repeat transposable element
ERVs: Endogenous retroviruses
MIC: Micronucleus
MAC: Macronucleus
HSFs: Heat shock factors

## References

[1] Ichiyanagi K, Saito K. The fifth Japanese meeting on biological function and evolution through interactions between hosts and transposable elements. Mob DNA-Uk. 2022;13:3. 10.1186/s13100-022-00261-7 PMC8756742 35027075

[2] Ichiyanagi K, Saito K. TE studies in japan: the fourth Japanese meeting on host-transposon interactions. Mob DNA-Uk. 2019;10:11. 10.1186/s13100-019-0154-7 PMC6419827 30923579

[3] Ichiyanagi K, Ikeda Y, Saito K. The sixth Japanese meeting on biological function and evolution through interactions between hosts and transposable elements. Mob DNA. 2023;14(1):22. 38087291 10.1186/s13100-023-00310-9 PMC10714607

[4] Ichiyanagi K. TE studies in japan: the third Japanese meeting on host-transposon interactions. Mob DNA-Uk. 2016;7:26. 10.1186/s13100-019-0154-7 PMC6419827 30923579

[5] Miura A, Yonebayashi S, Watanabe K, Toyama T, Shimada H, Kakutani T. Mobilization of transposons by a mutation abolishing full DNA methylation in Arabidopsis. Nature. 2001;411(6834):212–4. 11346800 10.1038/35075612

[6] Tsukahara S, Kobayashi A, Kawabe A, Mathieu O, Miura A, Kakutani T. Bursts of Retrotransposition reproduced in Arabidopsis. Nature. 2009;461(7262):423–6. 19734880 10.1038/nature08351

[7] Fu Y, Kawabe A, Etcheverry M, Ito T, Toyoda A, Fujiyama A, et al. Mobilization of a plant transposon by expression of the transposon-encoded anti-silencing factor. EMBO J. 2013;32(17):2407–17. 23900287 10.1038/emboj.2013.169 PMC3773815

[8] Hosaka A, Saito R, Takashima K, Sasaki T, Fu Y, Kawabe A, et al. Evolution of sequence-specific anti-silencing systems in Arabidopsis. Nat Commun. 2017;8(1):2161. 29255196 10.1038/s41467-017-02150-7 PMC5735166

[9] Sasaki T, Kato K, Hosaka A, Fu Y, Toyoda A, Fujiyama A, et al. Arms race between anti-silencing and RdDM in noncoding regions of transposable elements. EMBO Rep. 2023;24(8):e56678. 37272687 10.15252/embr.202256678 PMC10398659

[10] Naish M, Alonge M, Wlodzimierz P, Tock AJ, Abramson BW, Schmucker A, et al. The genetic and epigenetic landscape of the Arabidopsis centromeres. Science. 2021;374(6569):eabi7489. 34762468 10.1126/science.abi7489 PMC10164409

[11] Shimada A, Cahn J, Ernst E, Lynn J, Grimanelli D, Henderson I, et al. Retrotransposon addiction promotes centromere function via epigenetically activated small RNAs. Nat Plants. 2024;10(9):1304–16. 39223305 10.1038/s41477-024-01773-1 PMC11410651

[12] Tsukahara S, Bousios A, Perez-Roman E, Yamaguchi S, Leduque B, Nakano A, et al. Centrophilic retrotransposon integration via CENH3 chromatin in Arabidopsis. Nature. 2025;637(8046):744–8. 39743586 10.1038/s41586-024-08319-7 PMC11735389

[13] Kojima KK, Bao W. IS481EU shows a new connection between eukaryotic and prokaryotic DNA transposons. Biology (Basel). 2023;12(3):365. 10.3390/biology12030365 PMC10045372 36979057

[14] Kojima KK. Troyka represents a unique lineage of virus-like retroelements. J Gen Virol. 2025;106(9):002143. 10.1099/jgv.0.002143 PMC12440569 40952902

[15] Kojima KK. Helenus and Ajax, two groups of Non-Autonomous LTR Retrotransposons, represent a new type of small RNA Gene-Derived mobile elements. Biology (Basel). 2024;13(2):119. 10.3390/biology13020119 PMC10886601 38392337

[16] Brahmachari V, Kohli S, Gulati P. In praise of mealybugs. J Genet. 2018;97(2):379–89. 29932057

[17] Miyoshi T, Makino T, Moran JV. Poly(ADP-Ribose) polymerase 2 recruits replication protein A to sites of LINE-1 integration to facilitate Retrotransposition. Mol Cell. 2019;75(6):1286–e9812. 31473101 10.1016/j.molcel.2019.07.018 PMC6754305

[18] Luqman-Fatah A, Watanabe Y, Uno K, Ishikawa F, Moran JV, Miyoshi T. The interferon stimulated gene-encoded protein HELZ2 inhibits human LINE-1 Retrotransposition and LINE-1 RNA-mediated type I interferon induction. Nat Commun. 2023;14(1):203. 36639706 10.1038/s41467-022-35757-6 PMC9839780

[19] Luqman-Fatah A, Nishimori K, Amano S, Fumoto Y, Miyoshi T. Retrotransposon life cycle and its impacts on cellular responses. RNA Biol. 2024;21(1):11–27. 39396200 10.1080/15476286.2024.2409607 PMC11485995

[20] Tsukahara S, Kawabe A, Kobayashi A, Ito T, Aizu T, Shin-i T, et al. Centromere-targeted de Novo integrations of an LTR retrotransposon of Arabidopsis lyrata. Genes Dev. 2012;26(7):705–13. 22431508 10.1101/gad.183871.111 PMC3323881

[21] Hiraizumi M, Perry NT, Durrant MG, Soma T, Nagahata N, Okazaki S, et al. Structural mechanism of Bridge RNA-guided recombination. Nature. 2024;630(8018):994–1002. 38926616 10.1038/s41586-024-07570-2 PMC11208158

[22] Kato K, Okazaki S, Schmitt-Ulms C, Jiang K, Zhou W, Ishikawa J, et al. RNA-triggered protein cleavage and cell growth arrest by the type III-E CRISPR nuclease-protease. Science. 2022;378(6622):882–9. 36423304 10.1126/science.add7347 PMC11126364

[23] Kato K, Zhou W, Okazaki S, Isayama Y, Nishizawa T, Gootenberg JS, et al. Structure and engineering of the type III-E CRISPR-Cas7-11 effector complex. Cell. 2022;185(13):2324–e3716. 35643083 10.1016/j.cell.2022.05.003

[24] Nishimasu H, Shi X, Ishiguro S, Gao L, Hirano S, Okazaki S, et al. Engineered CRISPR-Cas9 nuclease with expanded targeting space. Science. 2018;361(6408):1259–62. 30166441 10.1126/science.aas9129 PMC6368452

[25] To TK, Kakutani T. Crosstalk among pathways to generate DNA methylome. Curr Opin Plant Biol. 2022;68:102248. 35724481 10.1016/j.pbi.2022.102248

[26] To TK, Nishizawa Y, Inagaki S, Tarutani Y, Tominaga S, Toyoda A, et al. RNA interference-independent reprogramming of DNA methylation in Arabidopsis. Nat Plants. 2020;6(12):1455–67. 33257860 10.1038/s41477-020-00810-z

[27] To TK, Yamasaki C, Oda S, Tominaga S, Kobayashi A, Tarutani Y, et al. Local and global crosstalk among heterochromatin marks drives DNA methylome patterning in Arabidopsis. Nat Commun. 2022;13(1):861. 35165291 10.1038/s41467-022-28468-5 PMC8844080

[28] Oda S, Tominaga S, Takeuchi S, Osakabe A, Kawabe A, Berger F et al. Antagonistic histone H2A variants and autonomous heterochromatin formation shape epigenomic patterns in Arabidopsis. bioRxiv. 2025:2025.10.19.683276. 10.1038/s41467-026-74770-x PMC13457619 42380212

[29] Naito K, Cho E, Yang G, Campbell MA, Yano K, Okumoto Y, et al. Dramatic amplification of a rice transposable element during recent domestication. Proc Natl Acad Sci U S A. 2006;103(47):17620–5. 17101970 10.1073/pnas.0605421103 PMC1693796

[30] Teramoto S, Tsukiyama T, Okumoto Y, Tanisaka T. Early embryogenesis-specific expression of the rice transposon Ping enhances amplification of the MITE mPing. PLoS Genet. 2014;10(6):e1004396. 24921928 10.1371/journal.pgen.1004396 PMC4055405

[31] Yamanaka S, Nishihara H, Toh H, Eijy Nagai LA, Hashimoto K, Park SJ, et al. Broad heterochromatic domains open in gonocyte development prior to de Novo DNA methylation. Dev Cell. 2019;51(1):21–34. e5. 31474564 10.1016/j.devcel.2019.07.023

[32] Uneme Y, Maeda R, Nakayama G, Narita H, Takeda N, Hiramatsu R, et al. Morc1 reestablishes H3K9me3 heterochromatin on piRNA-targeted transposons in gonocytes. Proc Natl Acad Sci U S A. 2024;121(13):e2317095121. 38502704 10.1073/pnas.2317095121 PMC10990106

[33] Iki T, Takami M, Kai T. Modulation of Ago2 loading by Cyclophilin 40 endows a unique repertoire of functional MiRNAs during sperm maturation in drosophila. Cell Rep. 2020;33(6):108380. 33176138 10.1016/j.celrep.2020.108380

[34] Iki T, Kawaguchi S, Kai T. miRNA/siRNA-directed pathway to produce noncoding PiRNAs from endogenous protein-coding regions ensures drosophila spermatogenesis. Sci Adv. 2023;9(29):eadh0397. 37467338 10.1126/sciadv.adh0397 PMC10355832

[35] Kai T, Zheng J, Iki T. Paralog-Dependent specialization of Paf1C Subunit, Ctr9, for sex chromosome gene regulation and male germline differentiation in drosophila. Genes Cells. 2025;30(5):e70040. 40763999 10.1111/gtc.70040 PMC12324932

[36] Chary S, Hayashi R. Pre-Cambrian origin of envelope-carrying retrotransposons in metazoans. eLife. 2025;14:RP108449. 10.7554/eLife.108449 PMC13524379 42663318

[37] Van Vu B, Nguyen Q, Kondo-Takeoka Y, Murata T, Kadotani N, Thi Nguyen G, et al. Copy number-dependent DNA methylation of the pyricularia oryzae MAGGY retrotransposon is triggered by DNA damage. Commun Biol. 2021;4(1):351. 33742058 10.1038/s42003-021-01836-5 PMC7979813

[38] Noto T, Mochizuki K. Whats, hows and whys of programmed DNA elimination in tetrahymena. Open Biol. 2017;7(10):170172. 10.1098/rsob.170172 PMC5666084 29021213

[39] Kataoka K, Mochizuki K. Phosphorylation of an HP1-like protein regulates heterochromatin body assembly for DNA elimination. Dev Cell. 2015;35(6):775–88. 26688337 10.1016/j.devcel.2015.11.017 PMC4695338

[40] Kataoka K, Noto T, Mochizuki K. Phosphorylation of an HP1-like protein is a prerequisite for heterochromatin body formation in tetrahymena DNA elimination. Proc Natl Acad Sci U S A. 2016;113(32):9027–32. 27466409 10.1073/pnas.1606012113 PMC4987781

[41] Peter CJ, Agarwal A, Watanabe R, Kassim BS, Wang X, Lambert TY, et al. Single chromatin fiber profiling and nucleosome position mapping in the human brain. Cell Rep Methods. 2024;4(12):100911. 39631398 10.1016/j.crmeth.2024.100911 PMC11704683

[42] Ohno T, Akase T, Kono S, Kurasawa H, Takashima T, Kaneko S, et al. Biallelic and gene-wide genomic substitution for endogenous intron and retroelement mutagenesis in human cells. Nat Commun. 2022;13(1):4219. 35864085 10.1038/s41467-022-31982-1 PMC9304424

[43] Ito H. Environmental stress and transposons in plants. Genes Genet Syst. 2022;97(4):169–75. 35922916 10.1266/ggs.22-00045

[44] Hasegawa R, Ito H. Transposition of the heat-activated retrotransposon ONSEN results in enhanced hypocotyl elongation. Genes Genet Syst. 2025;100:24-00110. 10.1266/ggs.24-00110 39864852

[45] Takei T, Tsukada M, Tamura K, Hara-Nishimura I, Fukao Y, Kurihara Y, et al. ARGONAUTE1-binding Tudor domain proteins function in small interfering RNA production for RNA-directed DNA methylation. Plant Physiol. 2024;195(2):1333–46. 38446745 10.1093/plphys/kiae135

